# Empirical Likelihood-Based ANOVA for Trimmed Means

**DOI:** 10.3390/ijerph13100953

**Published:** 2016-09-27

**Authors:** Mara Velina, Janis Valeinis, Luca Greco, George Luta

**Affiliations:** 1 Department of Mathematics, Faculty of Physics and Mathematics, University of Latvia, Riga LV-1002, Latvia; mara.velina@lu.lv; 2 Department of Law, Economics, Management and Quantitative Methods, University of Sannio, Benevento 82100, Italy; luca.greco@unisannio.it; 3 Department of Biostatistics, Bioinformatics and Biomathematics, Georgetown University, Washington, DC 20057, USA; George.Luta@georgetown.edu

**Keywords:** ANOVA, empirical likelihood, trimmed means, robust statistics, hypothesis testing

## Abstract

In this paper, we introduce an alternative to Yuen’s test for the comparison of several population trimmed means. This nonparametric ANOVA type test is based on the empirical likelihood (EL) approach and extends the results for one population trimmed mean from Qin and Tsao (2002). The results of our simulation study indicate that for skewed distributions, with and without variance heterogeneity, Yuen’s test performs better than the new EL ANOVA test for trimmed means with respect to control over the probability of a type I error. This finding is in contrast with our simulation results for the comparison of means, where the EL ANOVA test for means performs better than Welch’s heteroscedastic *F* test. The analysis of a real data example illustrates the use of Yuen’s test and the new EL ANOVA test for trimmed means for different trimming levels. Based on the results of our study, we recommend the use of Yuen’s test for situations involving the comparison of population trimmed means between groups of interest.

## 1. Introduction

The comparison of the means of several populations is frequently encountered in the statistical analysis of data from environmental research and public health studies. Typically, ANOVA is used to compare these means of interest, for example, for the comparison of means of blood lead levels between groups of children receiving different interventions. Practical situations may involve complications such as unbalanced designs (i.e., unequal sample sizes for the groups), variance heterogeneity, and departures from normality. It may be the case, for instance, that the distributions underlying the data from each group are truly heavy tailed or skewed, but it is also possible that such departures from normality are due to few observations located away from the bulk of the data in the tails of the distribution. It is well-known that the classical ANOVA *F* test cannot handle such violations of its assumptions, and, as a consequence, it has problems controlling the probability of the type I error at the specified nominal level. Heteroscedasticity and/or outliers can completely break down the results of the ANOVA *F* test when not properly taken into account (see, for example, [1]). Given this limitation of the ANOVA *F* test, there is a need for ANOVA type tests that are robust to both heteroscedasticity and outliers.

A statistical test that satisfies these requirements is the test developed by Yuen [2], who proposed a modified version of Welch’s heteroscedastic *F* test [3]. The latter test is designed to deal with heteroscedasticity for normally distributed data, and it is using the sample means and sample variances to estimate their population counterparts. Since the sample mean and the sample variance are not robust to outliers, Yuen [2] proposed to replace them with a pair of robust estimators consisting of the trimmed mean and the Winsorized variance. Such an approach provides a better control of the probability of the type I error for one-way ANOVA situations involving unbalanced designs and skewed distributions (see [4]). There are two important comments to be made. The first comment is that the construction of Yuen’s test has a somewhat ad hoc nature, by replacing the least squares estimators with robust versions. The second comment is that Yuen’s test is no longer a test for the comparison of populations means, but, rather, it is a test to compare population trimmed means. It may be preferable to make inferences regarding the population trimmed means rather than the population means when the underlying distributions for the groups are skewed, since the trimmed means are more representative for the bulk of the data in those situations [5].

In this paper, we present an alternative to Yuen’s test, a new nonparametric test that can be used to compare several trimmed means based on the empirical likelihood (EL) approach to statistical inference [6,7,8]. The EL method (see [9] for a detailed overview) is a popular nonparametric approach that does not require normality (or other distributional assumptions) and can be regarded as a data adaptive method. We develop an EL-based ANOVA test for the comparison of trimmed means that takes advantage of the nonparametric nature of the EL approach, by extending the results of Qin and Tsao [8] who introduced the EL method for a trimmed mean (see also the results from [10]). All technical details regarding the tests considered in this paper (including the asymptotic results for the new EL-based ANOVA for trimmed means) are provided in the Appendix A, Appendix B, Appendix C and Appendix D.

The paper is organized as follows. In Section 2, we present and interpret the results of a simulation study that compares the performance of the EL-based ANOVA for trimmed means and means with alternative methods under several scenarios involving skewed distributions. In Section 3, we analyze a real data set using different types of tests for the comparison of population trimmed means and population means. We end the paper by presenting conclusions in Section 4.

## 2. Simulation Study

For simplicity, we present only situations where we are interested in the comparison of three population trimmed means or three population means (k=3), while having samples of equal sizes. We consider scenarios involving skewed distributions, with and without variance heterogeneity. For the EL ANOVA for trimmed means, we consider only *symmetric* trimming, where all samples are trimmed symmetrically. We note that, although we are primarily interested in the performance of the tests for the comparison of trimmed means, EL ANOVA for trimmed means (panel *ELT*) and Yuen’s test (panel *Yuen*); for completeness purposes, we are also including the results for the tests for the comparison of means, specifically the classical ANOVA *F* test (panel *F test*), Welch’s heteroscedastic *F* test for means (panel *Welch*), and the EL ANOVA for means (panel *EL*). For Welch’s test and Yuen’s test, we have used the R function t1way (see Wilcox [11]). The *R* functions that provide the implementation of the EL ANOVA methods for trimmed means and means are available from the corresponding author upon request.

For the simulation study, we investigate the potential effect of the shape of the distributions on the estimated probability of type I errors. We consider several skewed distributions with and without variance heterogeneity. We use a simulation design similar to that from [5], where (trimmed) means of only two independent skewed populations are compared. For the scenario with homogeneous variances (scenario 1), we simulate data from three independent skewed distributions. We consider the $\chi_{3}^{2}$ distribution, the lognormal distribution with normal mean μ=0 and normal scale σ=1, the gamma distribution with shape parameter α=2 and scale parameter σ=1, and the skew-normal distribution with location parameter ξ=0, scale parameter ω=1, and slant parameter α=1 (see [12]). For the scenario with heterogeneous variances (scenario 2), we further transform the data simulated from the three independent skewed distributions as to have the ratios between variances to be either 1:4:9 or 1:1:36. To ensure that the relevant $H_{0}^{T}$ of equal trimmed means or $H_{0}$ of equal means are true, before altering the variances, we center the data using the theoretically determined trimmed means (when using tests for the comparison of trimmed means) or means (when using tests for the comparison of means). We use 10,000 Monte Carlo simulations to calculate the empirical probability of type I errors for the tests performed at the nominal 0.05 significance level.

Table 1 presents the empirical probability of type I errors for the different tests for the situation involving skewed distributions with homogeneous variances (scenario 1). Regarding the comparison of trimmed means, the results for Yuen’s test are closer to the nominal significance level than those for the EL ANOVA test for trimmed means. By contrast, among the tests that compare means, the results of the EL ANOVA test for means are closest to the nominal significance level. Table 2 and Table 3 present the corresponding results for the same tests for situations involving skewed distributions with heterogeneous variances (scenario 2). We note that it is more difficult to control the probability of a type I error when the ratios between variances are 1:1:36 than when they are 1:4:9. Similar to the homogeneous variances scenario, the results for the heterogeneous variances scenario suggest that Yuen’s test performs best among the tests for the comparison of trimmed means, while the EL ANOVA test performs best among the tests for the comparison of means.

## 3. Real Data Example

To illustrate the use of the EL ANOVA for trimmed means and means, we use the Oslo Transect data set [13]. This real data set includes 360 observations corresponding to different plants collected along a 120 km transect running through the city of Oslo, Norway. The concentrations of 25 chemical elements found in these plants were recorded together with factors that may influence the mineral concentration. Except for not including two chemical elements, *Au* and *Na*, this data is available within *R* package *rrcov* [14] as *OsloTransect* dataset. We analyze this dataset, and, thus, only 23 chemical elements are included in Table 4. To preserve the skewness of the data, we have also used the raw data, as opposed to the log transformed data (as done in [13]). After removing the observations with missing values, we are left with 332 observations. We consider the 23 concentrations of chemical elements as the response variables, and the lithology as a group variable with four levels.

As for the simulation study, even though our main interest is in tests that compare population trimmed means, for completeness purposes, we also provide the results from the tests that compare population means. We consider three symmetric trimming strategies similar to those used in the simulation study. The entries from Table 4 provide the *p*-values from the tests for the comparison of population means and population trimmed means. We note that, for each trimming strategy, the *p*-values from the EL ANOVA for trimmed means (panel *ELT*) and Yuen’s test (panel *Yuen*) are very similar. In addition, the *p*-values from the EL ANOVA for means (panel *EL*) and Welch’s heteroscedastic *F* test (panel *Welch*) are also very similar.

## 4. Conclusions

In this paper, we introduce a new nonparametric ANOVA type test for the comparison of population trimmed means. Although the new method is derived from the general principles of the empirical likelihood approach, versus the somewhat ad hoc nature of the derivation of Yuen’s test from Welch’s heteroscedastic *F* test, the results of our simulation study in situations involving skewed distributions indicate that, unless the sample sizes per group are very large, the new EL ANOVA method for trimmed means performs worse than Yuen’s test with respect to control over the probability of a type I error. This is in contrast with our simulation results for the comparison of means, where the EL ANOVA for means performs better than Welch’s heteroscedastic *F* test. The analysis of the real data example provides similar *p*-values for the new EL ANOVA method for trimmed means and the Yuen’s test for different trimming levels, and also similar *p*-values for the EL ANOVA and Welch’s heteroscedastic *F* test.

Based on these results, we recommend the use of Yuen’s test for situations, where the research question involves the comparison of population trimmed means between groups of interest. The choice of the specific trimming strategy is an important and complex issue, since different trimming strategies imply different null hypotheses being tested. As such, the selection of the trimming strategy should be based on subject matter reasons that take into account what is known by the experts about the data under investigation. Alternatively, in the absence of expert knowledge information, different trimming strategies could be used to evaluate the sensitivity of the results to the choice of the trimming strategy.

## Figures and Tables

**Table 1:** Empirical probability of type I error for various tests for the equality of means and trimmed means of three independent skewed distributions with homogeneous variances. For methods involving trimmed means, symmetric trimming at level $\alpha_{i}=\beta_{i}=c$, i=1,2,3 is used.

$\chi_{3}^{2}$									
				Trimming level					
				c=5%		c=10%		c=20%	
*n*	*F* test	Welch	EL	Yuen	ELT	Yuen	ELT	Yuen	ELT
20	0.047	0.076	0.052	0.050	0.079	0.050	0.080	0.049	0.090
30	0.048	0.070	0.054	0.054	0.055	0.053	0.075	0.054	0.079
40	0.044	0.062	0.052	0.049	0.063	0.048	0.063	0.050	0.069
50	0.048	0.058	0.049	0.046	0.047	0.047	0.060	0.049	0.067
100	0.049	0.055	0.051	0.050	0.056	0.050	0.056	0.049	0.056
200	0.051	0.055	0.053	0.050	0.053	0.051	0.053	0.051	0.056
500	0.051	0.049	0.049	0.048	0.050	0.050	0.051	0.051	0.052
**Lognormal** (μ=0,σ=1)									
				Trimming level					
				c=5%		c=10%		c=20%	
*n*	*F* test	Welch	EL	Yuen	ELT	Yuen	ELT	Yuen	ELT
20	0.044	0.073	0.047	0.040	0.069	0.040	0.070	0.040	0.081
30	0.045	0.069	0.050	0.048	0.049	0.046	0.068	0.046	0.072
40	0.044	0.063	0.049	0.046	0.062	0.046	0.062	0.045	0.066
50	0.045	0.065	0.054	0.049	0.048	0.047	0.059	0.046	0.062
100	0.049	0.059	0.055	0.049	0.055	0.049	0.057	0.049	0.057
200	0.050	0.053	0.050	0.045	0.048	0.046	0.049	0.048	0.052
500	0.051	0.053	0.052	0.051	0.052	0.050	0.050	0.052	0.054
**Gamma** (α=2,σ=1)									
				Trimming level					
				c=5%		c=10%		c=20%	
*n*	*F* test	Welch	EL	Yuen	ELT	Yuen	ELT	Yuen	ELT
20	0.052	0.078	0.050	0.050	0.077	0.052	0.079	0.052	0.096
30	0.049	0.069	0.053	0.052	0.053	0.050	0.070	0.052	0.080
40	0.050	0.062	0.052	0.052	0.064	0.052	0.067	0.053	0.074
50	0.050	0.060	0.051	0.048	0.048	0.050	0.062	0.052	0.069
100	0.052	0.057	0.052	0.051	0.057	0.050	0.056	0.048	0.056
200	0.052	0.056	0.053	0.052	0.055	0.053	0.055	0.052	0.055
500	0.049	0.052	0.051	0.050	0.051	0.049	0.051	0.051	0.052
**Skew-normal** (ξ=0,ω=1,α=1)									
				Trimming level					
				c=5%		c=10%		c=20%	
*n*	*F* test	Welch	EL	Yuen	ELT	Yuen	ELT	Yuen	ELT
20	0.055	0.077	0.049	0.049	0.077	0.051	0.083	0.054	0.099
30	0.048	0.065	0.050	0.051	0.051	0.050	0.068	0.051	0.078
40	0.049	0.061	0.049	0.049	0.062	0.051	0.065	0.051	0.073
50	0.051	0.058	0.049	0.049	0.050	0.050	0.061	0.052	0.071
100	0.055	0.055	0.052	0.051	0.056	0.050	0.056	0.052	0.060
200	0.052	0.051	0.049	0.050	0.052	0.051	0.054	0.052	0.056
500	0.046	0.048	0.047	0.047	0.048	0.048	0.049	0.048	0.049

**Table 2:** Empirical probability of type I error for various tests for the equality of means and trimmed means of three independent skewed distributions with the ratios between variances being 1:4:9. For methods involving trimmed means, symmetric trimming at level $\alpha_{i}=\beta_{i}=c$, i=1,2,3 is used.

$\chi_{3}^{2}$									
				Trimming level					
				c=5%		c=10%		c=20%	
*n*	*F* test	Welch	EL	Yuen	ELT	Yuen	ELT	Yuen	ELT
20	0.086	0.101	0.071	0.064	0.096	0.061	0.094	0.062	0.109
30	0.083	0.088	0.071	0.060	0.062	0.064	0.084	0.064	0.091
40	0.080	0.075	0.062	0.060	0.072	0.055	0.072	0.054	0.078
50	0.079	0.067	0.057	0.050	0.051	0.051	0.066	0.052	0.071
100	0.079	0.063	0.058	0.055	0.060	0.055	0.060	0.054	0.062
200	0.085	0.060	0.057	0.057	0.059	0.054	0.058	0.054	0.058
500	0.073	0.052	0.051	0.053	0.054	0.052	0.054	0.051	0.053
**Lognormal** (μ=0,σ=1)									
				Trimming level					
				c=5%		c=10%		c=20%	
*n*	*F* test	Welch	EL	Yuen	ELT	Yuen	ELT	Yuen	ELT
20	0.110	0.146	0.113	0.078	0.106	0.070	0.106	0.066	0.114
30	0.109	0.131	0.111	0.063	0.064	0.065	0.088	0.063	0.090
40	0.100	0.115	0.100	0.066	0.081	0.062	0.083	0.059	0.084
50	0.098	0.110	0.099	0.060	0.060	0.060	0.074	0.059	0.077
100	0.097	0.090	0.083	0.058	0.063	0.055	0.062	0.055	0.063
200	0.082	0.071	0.069	0.051	0.055	0.052	0.055	0.054	0.058
500	0.077	0.061	0.060	0.051	0.053	0.052	0.053	0.051	0.054
**Gamma** (α=2,σ=1)									
				Trimming level					
				c=5%		c=10%		c=20%	
*n*	*F* test	Welch	EL	Yuen	ELT	Yuen	ELT	Yuen	ELT
20	0.086	0.099	0.070	0.061	0.093	0.062	0.097	0.065	0.111
30	0.083	0.080	0.063	0.058	0.062	0.056	0.079	0.060	0.090
40	0.079	0.074	0.061	0.058	0.073	0.056	0.073	0.059	0.083
50	0.079	0.068	0.057	0.049	0.050	0.053	0.066	0.058	0.075
100	0.078	0.059	0.055	0.053	0.060	0.053	0.060	0.053	0.061
200	0.078	0.057	0.054	0.053	0.055	0.053	0.057	0.053	0.057
500	0.079	0.052	0.050	0.049	0.051	0.050	0.050	0.052	0.053
**Skew-normal** (ξ=0,ω=1,α=1)									
				Trimming level					
				c=5%		c=10%		c=20%	
*n*	*F* test	Welch	EL	Yuen	ELT	Yuen	ELT	Yuen	ELT
20	0.080	0.079	0.048	0.048	0.081	0.052	0.085	0.054	0.106
30	0.075	0.069	0.050	0.051	0.054	0.053	0.073	0.057	0.085
40	0.080	0.060	0.049	0.050	0.064	0.050	0.069	0.053	0.075
50	0.076	0.060	0.050	0.050	0.051	0.051	0.064	0.053	0.073
100	0.079	0.053	0.049	0.048	0.054	0.048	0.055	0.051	0.059
200	0.078	0.054	0.051	0.051	0.054	0.051	0.054	0.052	0.056
500	0.075	0.049	0.047	0.048	0.049	0.048	0.049	0.048	0.049

**Table 3:** Empirical probability of type I error for various tests for the equality of means and trimmed means of three independent skewed distributions with the ratios between variances being 1:1:36. For methods involving trimmed means, symmetric trimming at level $\alpha_{i}=\beta_{i}=c$, i=1,2,3 is used.

$\chi_{3}^{2}$									
				Trimming level					
				c=5%		c=10%		c=20%	
*n*	*F* test	Welch	EL	Yuen	ELT	Yuen	ELT	Yuen	ELT
20	0.124	0.090	0.067	0.059	0.087	0.056	0.088	0.056	0.102
30	0.119	0.080	0.064	0.056	0.058	0.058	0.078	0.058	0.086
40	0.116	0.070	0.058	0.055	0.067	0.053	0.067	0.052	0.072
50	0.112	0.063	0.052	0.048	0.049	0.051	0.063	0.052	0.069
100	0.111	0.061	0.055	0.053	0.059	0.051	0.058	0.050	0.058
200	0.113	0.059	0.056	0.054	0.057	0.053	0.056	0.052	0.056
500	0.102	0.053	0.053	0.053	0.054	0.050	0.052	0.050	0.052
**Lognormal** (μ=0,σ=1)									
				Trimming level					
				c=5%		c=10%		c=20%	
*n*	*F* test	Welch	EL	Yuen	ELT	Yuen	ELT	Yuen	ELT
20	0.168	0.126	0.101	0.071	0.095	0.062	0.095	0.058	0.103
30	0.166	0.118	0.098	0.055	0.056	0.060	0.081	0.057	0.084
40	0.153	0.104	0.089	0.060	0.076	0.061	0.077	0.057	0.080
50	0.148	0.095	0.086	0.052	0.053	0.056	0.068	0.055	0.072
100	0.136	0.080	0.075	0.054	0.061	0.053	0.062	0.054	0.061
200	0.119	0.064	0.062	0.050	0.054	0.049	0.052	0.051	0.055
500	0.112	0.056	0.055	0.052	0.053	0.050	0.051	0.052	0.054
**Gamma** (α=2,σ=1)									
				Trimming level					
				c=5%		c=10%		c=20%	
*n*	*F* test	Welch	EL	Yuen	ELT	Yuen	ELT	Yuen	ELT
20	0.123	0.089	0.064	0.058	0.089	0.059	0.093	0.060	0.107
30	0.122	0.079	0.061	0.057	0.059	0.055	0.077	0.058	0.086
40	0.116	0.071	0.057	0.056	0.069	0.054	0.070	0.055	0.077
50	0.113	0.066	0.055	0.051	0.052	0.051	0.064	0.053	0.070
100	0.110	0.059	0.053	0.053	0.058	0.052	0.057	0.051	0.060
200	0.109	0.054	0.051	0.051	0.054	0.050	0.054	0.051	0.055
500	0.108	0.052	0.051	0.049	0.050	0.050	0.051	0.050	0.051
**Skew-normal** (ξ=0,ω=1,α=1)									
				Trimming level					
				c=5%		c=10%		c=20%	
*n*	*F* test	Welch	EL	Yuen	ELT	Yuen	ELT	Yuen	ELT
20	0.113	0.077	0.050	0.047	0.080	0.049	0.083	0.054	0.103
30	0.107	0.067	0.051	0.050	0.052	0.052	0.074	0.054	0.083
40	0.112	0.060	0.049	0.049	0.063	0.051	0.067	0.054	0.074
50	0.107	0.058	0.048	0.049	0.050	0.051	0.064	0.055	0.074
100	0.107	0.054	0.048	0.048	0.054	0.049	0.056	0.052	0.060
200	0.106	0.053	0.051	0.052	0.054	0.051	0.054	0.053	0.057
500	0.106	0.048	0.048	0.050	0.051	0.050	0.051	0.049	0.051

**Table 4:** *p*-values from tests of equality of means and trimmed means of 23 chemical element concentrations in plants collected along the Oslo Transect ([13]). Symmetric trimming, $\alpha_{i}=\beta_{i}=c$, i=1,…,4.

						Trimming Level			
				c=5%		c=10%		c=20%	
Element	F Test	Welch	EL	Yuen	ELT	Yuen	ELT	Yuen	ELT
Ag	0.26	0.10	0.09	0.22	0.23	0.42	0.41	0.74	0.73
B	0.08	0.09	0.07	0.10	0.09	0.12	0.11	0.18	0.16
Ba	0.01	0.01	0.01	0.03	0.03	0.02	0.02	<0.01	<0.01
Ca	0.15	0.19	0.18	0.22	0.22	0.31	0.31	0.42	0.41
Cd	0.08	0.05	0.04	0.09	0.09	0.05	0.05	0.03	0.02
Co	<0.01	0.01	0.01	<0.01	<0.01	<0.01	<0.01	<0.01	<0.01
Cr	0.17	<0.01	<0.01	<0.01	<0.01	<0.01	<0.01	<0.01	<0.01
Cu	0.44	0.26	0.24	0.66	0.67	0.77	0.76	0.76	0.75
Fe	0.03	0.02	0.01	0.04	0.04	0.02	0.02	0.04	0.03
Hg	0.31	0.29	0.27	0.35	0.37	0.19	0.18	0.40	0.38
K	0.47	0.28	0.26	0.50	0.52	0.53	0.52	0.58	0.57
La	0.28	<0.01	<0.01	0.01	0.01	0.13	0.10	0.01	0.01
Mg	0.24	0.23	0.21	0.28	0.28	0.38	0.37	0.57	0.56
Mn	<0.01	<0.01	<0.01	<0.01	<0.01	<0.01	<0.01	<0.01	<0.01
Mo	0.02	<0.01	<0.01	0.02	0.02	0.04	0.03	0.17	0.15
Ni	<0.01	<0.01	<0.01	<0.01	<0.01	<0.01	<0.01	0.02	0.01
P	0.28	0.25	0.24	0.39	0.40	0.43	0.43	0.58	0.57
Pb	0.52	<0.01	<0.01	0.01	0.01	0.01	0.01	<0.01	<0.01
S	0.58	0.55	0.54	0.70	0.72	0.78	0.78	0.81	0.81
Sb	0.16	0.01	<0.01	0.21	0.22	0.19	0.19	0.25	0.20
Sr	0.14	0.07	0.06	0.18	0.19	0.22	0.22	0.10	0.09
Ti	0.01	0.01	<0.01	0.06	0.06	0.09	0.08	0.08	0.07
Zn	0.88	0.80	0.79	0.97	0.97	0.97	0.97	0.97	0.96

## Appendix A. Statistical Tests Not Based on EL

Let $Y_{i}=\left(Y_{i1},Y_{i2},\ldots ,Y_{in_{i}}\right),i=1,2,\ldots ,k$, be independent random samples from *k* different distributions with population means $\mu_{i}$. We are interested in testing the null hypothesis of equal population means

(A1) $$H_{0}:\mu_{1}=\ldots =\mu_{k}=\mu .\;$$

Under the assumption of equal variances (homoscedasticity) and normally distributed data in each group, i.e., $Y_{ij}∼N\left(\mu_{i},\sigma \right)$, one can use the classical ANOVA *F* test

$$F=\frac{\sum_{i=1}^{k}n_{i}{\left({\bar{Y}}_{i\cdot }-{\bar{Y}}_{\cdot \cdot }\right)}^{2}/\left(k-1\right)}{\sum_{i=1}^{k}\left(n_{i}-1\right)s_{i}^{2}/\left(N-k\right)},$$

where

$${\bar{Y}}_{i\cdot }=\frac{1}{n_{i}}\sum_{j=1}^{n_{i}}Y_{ij}\;\mathrm{and}\;s_{i}^{2}=\frac{1}{n_{i}-1}\sum_{j=1}^{n_{i}}{\left(Y_{ij}-{\bar{Y}}_{i\cdot }\right)}^{2}$$

are the sample mean and sample variance of the *i*-th group, respectively, and

$${\bar{Y}}_{\cdot \cdot }=\sum_{i=1}^{k}\sum_{j=1}^{n_{i}}Y_{ij}/N$$

is the pooled sample mean. The null hypothesis in (A1) is rejected at level *c*, if $F>F_{c,k-1,N-k}$, where $F_{c,k-1,N-k}$ is the critical value based on the *F* distribution with k−1 and N−k degrees of freedom.

Let us suppose now that $Y_{ij}∼N\left(\mu_{i},\sigma_{i}\right)$ for i=1,…,k. Welch’s heteroscedastic *F* test [3] is designed to be robust to the violation of the assumption of equal group variances. The main difference with the classical ANOVA *F* test is that the following weights are used:

$$w_{i}=\frac{n_{i}}{s_{i}^{2}}.$$

The Welch’s heteroscedastic *F* test statistics is defined by

$$F_{W}=A\frac{\sum_{i=1}^{k}w_{i}{\left({\bar{Y}}_{i\cdot }-{\bar{Y}}^{\prime }\right)}^{2}}{k-1},$$

where

$${\bar{Y}}^{\prime }=\frac{\sum_{i=1}^{k}w_{i}{\bar{Y}}_{i\cdot }}{\sum_{i=1}^{k}w_{i}},$$

$$A={\left(1+\frac{2(k-2)}{\left(k^{2}-1\right)}\sum_{i=1}^{k}\left(\frac{1}{n_{i}-1}\right){\left(1-\frac{w_{i}}{\sum_{i=1}^{k}w_{i}}\right)}^{2}\right)}^{-1}.$$

The null hypothesis (A1) is rejected at level *c* if $F_{W}\geq F_{c,k-1,\nu_{W}}$, where

$$\nu_{W}={\left[\frac{3}{k^{2}-1}\sum_{i=1}^{k}\left(\frac{1}{n_{i}-1}\right){\left(1-\frac{w_{i}}{\sum_{i=1}^{k}w_{i}}\right)}^{2}\right]}^{-1}.$$

Yuen’s test, i.e., the robust modification of Welch’s heteroscedastic *F* test, is designed to be robust to departures from normality. The test is obtained by using the sample trimmed means and Winsorized variances instead of the sample means and variances. Let $Y_{i(1)},Y_{i(2)},\ldots ,Y_{i(n_{i})}$ denote the order statistics for the *i*th sample. Let $q_{i}=\left[n_{i}\alpha_{i}\right]+1$ and $r_{i}=n_{i}-\left[n_{i}\beta_{i}\right]$, where $0<\alpha_{i}<1/2$ and $0<\beta_{i}<1/2$ represent the proportion of observations trimmed from the left and from the right tail of the distribution, respectively, and [x] denotes the largest integer less than or equal to *x*. Then, $m_{i}=n_{i}-\left[n_{i}\alpha_{i}\right]-\left[n_{i}\beta_{i}\right]$ represents the effective sample size after trimming and the sample trimmed mean of the *i*th group is defined as

$${\bar{Y}}_{\alpha \beta i}=\frac{1}{m_{i}}\sum_{j=q_{i}}^{r_{i}}Y_{i(j)}\;.$$

Let $W_{ij}$ represent the new observations after replacing the trimmed observations in the lower and upper tails with the lowest and highest untrimmed values of the sample, i.e.,

$$W_{ij}=\left\{\begin{array}{cc} Y_{i(q_{i})}, & Y_{ij}\leq Y_{i(q_{i})},\\ Y_{ij}, & Y_{i(q_{i})}<Y_{ij}<Y_{i(r_{i})},\\ Y_{i(r_{i})}, & Y_{ij}\geq Y_{i(r_{i})}. \end{array}\right.$$

The sample Winsorized variance for the *i*-th group is computed as

$$s_{wi}^{2}=\frac{\sum_{j=1}^{n_{i}}{\left(W_{ij}-{\bar{Y}}_{wi}\right)}^{2}}{n_{i}-1},$$

where

$${\bar{Y}}_{wi}=\frac{1}{n}\sum_{j=1}^{n_{i}}W_{ij}.$$

Yuen’s test statistics is given by

$$F_{YT}=A_{T}\frac{\sum_{i=1}^{k}w_{iT}{\left({\bar{Y}}_{\alpha \beta i}-\tilde{Y}\right)}^{2}}{k-1},$$

where

$$w_{iT}=\frac{m_{i}\left(m_{i}-1\right)}{\left(n_{i}-1\right)s_{wi}^{2}},$$

$$\tilde{Y}=\frac{\sum_{i=1}^{k}w_{iT}{\bar{Y}}_{\alpha \beta i}}{\sum_{i=1}^{k}w_{iT}},$$

and

$$A_{T}={\left(1+\frac{2(k-2)}{\left(k^{2}-1\right)}\sum_{i=1}^{k}\left(\frac{1}{m_{i}-1}\right){\left(1-\frac{w_{iT}}{\sum_{i=1}^{k}w_{iT}}\right)}^{2}\right)}^{-1}.$$

The null hypothesis of equal (trimmed) means is rejected at level *c* if $F_{YT}\geq F_{c,k-1,\nu_{YT}}$, where

$$\nu_{YT}={\left[\frac{3}{k^{2}-1}\sum_{i=1}^{k}\left(\frac{1}{m_{i}-1}\right){\left(1-\frac{w_{iT}}{\sum_{i=1}^{k}w_{iT}}\right)}^{2}\right]}^{-1}.$$

Note that this test reduces to Welch’s heteroscedastic *F* test when there is no trimming.

## Appendix B. EL-Based ANOVA for Means

Let $F_{i}$ denote a candidate for the true unknown distribution $F_{i0}$ and $v_{ij}=F_{i}\left\{Y_{ij}\right\}$ denote the jump of $F_{i}$ at $\left\{Y_{ij}\right\}$. The EL for the *i*-th sample is $L\left(F_{i}\right)=\prod_{j=1}^{n_{i}}v_{ij}$ and corresponds to a multinomial distribution defined on the *i*-th sample by attaching a weight $v_{ij}$ to each $Y_{ij}$. The weights $v_{ij}=v_{ij}\left(\mu_{i}\right)$ satisfy the conditions

1. $v_{ij}\geq 0;$
2. $\sum_{j=1}^{n_{i}}v_{ij}=1;$
3. $\sum_{j=1}^{n_{i}}v_{ij}Y_{ij}=\mu_{i}.$

The function $L(F_{i})$ attains its maximum value when $v_{ij}=n_{i}^{-1}$. Similar to the classical approach based on the parametric likelihood, the profile EL ratio function is defined as

$$R\left(\mu_{i}\right)=\underset{v_{ij}}{\sup}\left\{\prod_{j=1}^{n_{i}}n_{i}v_{ij},\sum_{j=1}^{n_{i}}v_{ij}=1,\sum_{j=1}^{n_{i}}v_{ij}\left(Y_{ij}-\mu_{i}\right)=0\right\}.$$

For an ANOVA model, Owen ([15]) defined the *k*-sample EL as the product of *k* group specific empirical likelihoods. Therefore, given the *k* samples, the profile EL ratio function can be defined as follows:

(B1) $$R\left(\mu \right)=\prod_{i=1}^{k}R_{i}\left(\mu \right)=\underset{v_{ij}}{\sup}\left\{\prod_{i=1}^{k}\prod_{j=1}^{n_{i}}n_{i}v_{ij}:\sum_{j=1}^{n_{i}}v_{ij}=1,\sum_{j=1}^{n_{i}}v_{ij}\left(Y_{ij}-\mu \right)=0\right\},$$

where $v_{ij}=v_{ij}\left(\mu \right)$. Under the null hypothesis (A1), the k−1 contrasts between means are constrained to be zero, and if $\mu =\mu_{0}+O\left(n_{0}^{-1/2}\right)$, where $\mu_{0}$ is the true unknown common mean and $n_{0}=\min_{1\leq i\leq k}n_{i}$, then

(B2) $$-2\log\max_{\mu }R\left(\mu \right)=\sum_{i=1}^{k}w_{i}{\left({\bar{Y}}_{i\cdot }-\bar{Y}\right)}^{2}+O_{p}\left(n_{0}^{-1/2}\right)\overset{d}{\to }\chi_{\left(k-1\right)}^{2}$$

as $n_{0}\to \infty$, where ${\bar{Y}}_{i\cdot }$ is the sample mean for the *i*-th sample, $\bar{Y}$ is the common mean estimator

$$\bar{Y}=\frac{\sum_{i=1}^{k}{\bar{Y}}_{i\cdot }w_{i}}{\sum_{i=1}^{k}w_{i}},$$

and the weights $w_{i}$ are inverse proportional with the sample variances $S_{i}^{2}$, i.e.,

$$w_{i}=\frac{n_{i}}{S_{i}^{2}}.$$

It is important to note that EL-based ANOVA is robust to heteroscedasticity (see [16]).

## Appendix C. EL for the Trimmed Mean

In a one sample situation, let $Y_{1},Y_{2},\ldots ,Y_{n}$ be independent identically distributed with $Y_{1}∼F_{0}$ and let $Y_{\left(1\right)},Y_{\left(2\right)},\ldots ,Y_{\left(n\right)}$ be the respective order statistics. Let q=[nα]+1 and r=n−[nβ], where 0<α<1/2 and 0<β<1/2, represent the proportion of observations trimmed from the left and right tails, respectively, and [x] denotes the largest integer less than or equal to *x*. Then, m=n−[nα]−[nβ] is the effective sample size after trimming. Let weights $p_{j}=0$ for j<q and j>r, $p_{j}\geq 0$ for q≤j≤r, and $\sum_{j=q}^{r}p_{j}=1$. Then, the profile EL ratio function for the trimmed sample is defined as

$$R\left(\mu_{\alpha \beta }\right)=\underset{p_{j}}{\sup}\{\prod_{j=q}^{r}mp_{j}:p_{j}\geq 0,\sum_{j=q}^{r}p_{j}=1,\sum_{j=q}^{r}p_{j}Y_{\left(j\right)}=\mu_{\alpha \beta }\}.$$

**Theorem** **C1.** *(Qin and Tsao, [8]) Let $\mu_{\alpha \beta }^{0}$ be the true value of $\mu_{\alpha \beta }$. Assume $F_{0}$ is continuous, $F_{0}^{\prime }\left(\xi_{\alpha }\right)>0$ and $F_{0}^{\prime }\left(\xi_{\beta }\right)>0$. Then,*

$$-2a\log R\left(\mu_{\alpha \beta }\right)\overset{d}{\to }\chi_{1}^{2},$$

*where*

$$a=\sigma_{\alpha \beta }^{2}/\left(\left(1-\alpha -\beta \right)\tau_{\alpha \beta }^{2}\right),$$

$$\sigma_{\alpha \beta }^{2}=\frac{1}{\left(1-\alpha -\beta \right)}\int_{\xi_{\alpha }}^{\xi_{1-\beta }}y^{2}dF_{0}\left(y\right)-\mu_{\alpha \beta }^{2},$$

$$\begin{array}{c} \tau_{\alpha \beta }^{2}=\frac{1}{{\left(1-\alpha -\beta \right)}^{2}}(\left(1-\alpha -\beta \right)\sigma_{\alpha \beta }^{2}+\beta \left(1-\beta \right){\left(\xi_{1-\beta }-\mu_{\alpha \beta }\right)}^{2}\\ -2\alpha \beta \left(\xi_{\alpha }-\mu_{\alpha \beta }\right)\left(\xi_{1-\beta }-\mu_{\alpha \beta }\right)+\alpha \left(1-\alpha \right){\left(\xi_{\alpha }-\mu_{\alpha \beta }\right)}^{2}). \end{array}$$

The unknown scaling constant *a* can be estimated consistently via $\hat{a}={\hat{\sigma }}_{\alpha \beta }^{2}/\left(\left(1-\alpha -\beta \right){\hat{\tau }}_{\alpha \beta }^{2}\right)$, where

$${\hat{\sigma }}_{\alpha \beta }^{2}=\frac{1}{\left(1-\alpha -\beta \right)}\int_{{\hat{\xi }}_{\alpha }}^{{\hat{\xi }}_{1-\beta }}y^{2}dF_{n}\left(y\right)-{\bar{Y}}_{\alpha \beta }^{2},$$

$$\begin{array}{c} {\hat{\tau }}_{\alpha \beta }^{2}=\frac{1}{{\left(1-\alpha -\beta \right)}^{2}}(\left(1-\alpha -\beta \right){\hat{\sigma }}_{\alpha \beta }^{2}+\beta \left(1-\beta \right){\left({\hat{\xi }}_{1-\beta }-{\bar{Y}}_{\alpha \beta }\right)}^{2}\\ \;-2\alpha \beta \left({\hat{\xi }}_{\alpha }-{\bar{Y}}_{\alpha \beta }\right)\left({\hat{\xi }}_{1-\beta }-{\bar{Y}}_{\alpha \beta }\right)+\alpha \left(1-\alpha \right){\left({\hat{\xi }}_{\alpha }-{\bar{Y}}_{\alpha \beta }\right)}^{2}), \end{array}$$

where ${\hat{\xi }}_{p}=\inf\left\{y:F_{n}\left(y\right)\geq p\right\}$ for any 0<p<1, and $F_{n}\left(y\right)$ is the empirical distribution function.

## Appendix D. EL-Based ANOVA for Trimmed Means

The ELRT (B2) is still sensitive to outliers. Following the approach from [8], one could develop a version of (B2) with trimmed means. The main result stated in [8] is given in Appendix C.

Let $Y_{i(1)},Y_{i(2)},\ldots ,Y_{i(n_{i})}$ denote the ordered *i*-th sample, set $q_{i}=\left[n_{i}\alpha_{i}\right]+1$ and $r_{i}=n_{i}-\left[n_{i}\beta_{i}\right]$, where $0<\alpha_{i}<1/2$ and $0<\beta_{i}<1/2$ represent the proportion of observations trimmed from the left and the right tails, respectively, and [x] denotes the largest integer less than or equal to *x*. Then, $m_{i}=n_{i}-\left[n_{i}\alpha_{i}\right]-\left[n_{i}\beta_{i}\right]$ is the effective sample size after trimming in each group. The group specific trimmed means and variances are

$$\begin{array}{ccc} {\bar{Y}}_{\alpha \beta i} & = & \frac{1}{m_{i}}\sum_{j=q_{i}}^{r_{i}}Y_{i(j)},\\ S_{\alpha \beta i}^{2} & = & m_{i}^{-1}\sum_{j=q_{i}}^{r_{i}}{\left(Y_{i(j)}-{\bar{Y}}_{\alpha \beta i}\right)}^{2}. \end{array}$$

Similar to (B1), define the profile EL ratio function over the trimmed samples, that is, as if the $m_{i}$ observations in each sample are independent, i.e.,

(D1) $$R\left(\mu_{\alpha \beta }\right)=\prod_{i=1}^{k}R_{i}\left(\mu_{\alpha \beta i}\right)=\underset{v_{ij}}{\sup}\left\{\prod_{i=1}^{k}\prod_{j=q_{i}}^{r_{i}}m_{i}v_{ij},\sum_{j=q_{i}}^{r_{i}}v_{ij}=1,\sum_{j=q_{1}}^{r_{1}}v_{ij}\left(Y_{i(j)}-\mu_{\alpha \beta }\right)=0\right\}.$$

It is important to note that the weights are no longer a function of the common population mean but of the common population trimmed mean, that is $v_{ij}=v_{ij}\left(\mu_{\alpha \beta }\right)$. As a consequence, we will obtain an ELRT for a different null hypothesis claiming the equality of population trimmed means (see [8,10]), that is

(D2) $$H_{0}^{T}:\mu_{\alpha \beta 1}=\mu_{\alpha \beta 2}=\ldots =\mu_{\alpha \beta k}=\mu_{\alpha \beta }\;.$$

When the underlying distribution of the data in each group is symmetric, the two hypotheses (A1) and (D2) are equivalent if symmetric trimming is performed. This equivalence does not hold for skewed distributions, for which it may be preferable to compare trimmed means rather than means [5]. The following result holds.

**Theorem** **D1.** *Let $\mu_{\alpha \beta 0}$ be the common population trimmed mean. Assume that $F_{i0}$ is continuous, $F_{i0}^{\prime }\left(\xi_{\alpha }\right)>\;0$ and $F_{i0}^{\prime }\left(\xi_{\beta }\right)>0$ for each i=1,…,k. If $\mu_{\alpha \beta i}=\mu_{\alpha \beta 0}+O_{p}\left(m_{0}^{-1/2}\right)$, i=1,2…,k, where $m_{0}=\min_{1\leq i\leq k}m_{i}$, then*

(D3) $$-2\sum_{i=1}^{k}a_{i}\max_{\mu }\log R_{i}\left(\mu_{\alpha \beta }\right)=\sum_{i=1}^{k}a_{i}w_{\alpha \beta i}{\left({\bar{Y}}_{\alpha \beta i}-{\bar{Y}}_{\alpha \beta }\right)}^{2}+O_{p}\left(m_{0}^{-1/2}\right)\overset{d}{\to }\chi_{\left(k-1\right)}^{2},$$

*where*

$$\begin{array}{ccc} {\bar{Y}}_{\alpha \beta } & = & \frac{\sum_{i=1}^{k}{\bar{Y}}_{\alpha \beta i}w_{\alpha \beta i}}{\sum_{i=1}^{k}w_{\alpha \beta i}}+o_{p}\left(m_{0}^{-1/2}\right),\\ w_{\alpha \beta i} & = & \frac{m_{i}}{S_{\alpha \beta i}^{2}}, \end{array}$$

*and the scaling factors are given by*

(D4) $$a_{i}=\sigma_{\alpha \beta i}^{2}/\left(\left(1-\alpha_{i}-\beta_{i}\right)\tau_{\alpha \beta i}^{2}\right)\;.$$

The quantities $\sigma_{\alpha \beta i}^{2}$ and $\tau_{\alpha \beta i}^{2}$ for the ith trimmed sample are defined in Appendix C.

**Proof.** By a Lagrange multiplier argument, it can be shown (see, for example, [9]), that the $v_{ij},i=1,2,\ldots ,k$ that maximize $R(\mu_{\alpha \beta })$ are given by

(D5) $$v_{ij}=\frac{1}{m_{i}\left(1+\lambda_{i}\left(Y_{i(j)}-\mu_{\alpha \beta }\right)\right)},j=q_{i},\ldots ,r_{i},$$

where the Lagrange multiplier $\lambda_{i}$ is the solution to

$$\sum_{j=q_{i}}^{r_{i}}\frac{Y_{i(j)}-\mu_{\alpha \beta }}{1+\lambda_{i}\left(Y_{i(j)}-\mu_{\alpha \beta }\right)}=0,$$

and

$$\lambda_{i}=\frac{{\bar{Y}}_{\alpha \beta i}-\mu_{\alpha \beta }}{\frac{1}{m_{i}}\sum_{j=q_{i}}^{r_{i}}{\left(Y_{i(j)}-\mu_{\alpha \beta }\right)}^{2}}+o_{p}\left(m_{i}^{-1/2}\right).$$

Then, by substituting $v_{ij}$ from (D5) in the expression (D1), we obtain

$$-2\log R\left(\mu_{\alpha \beta }\right)=2\sum_{i=1}^{k}\sum_{j=q_{i}}^{r_{i}}\log\left(1+\lambda_{i}\left(Y_{i(j)}-\mu_{\alpha \beta }\right)\right).$$

The maximum empirical likelihood estimator ${\bar{Y}}_{\alpha \beta }$ is the solution to $\partial R\left(\mu_{\alpha \beta }\right)/\partial \mu_{\alpha \beta }=0$:

$$\frac{\partial R(\mu_{\alpha \beta })}{\partial \mu_{\alpha \beta }}=-\sum_{i=1}^{k}\sum_{j=q_{i}}^{r_{i}}\left(\frac{\partial \lambda_{i}}{\partial \mu_{\alpha \beta }}\left(Y_{i(j)}-\mu_{\alpha \beta }\right)-\lambda_{i}\right){\left(1+\lambda_{i}\left(Y_{i(j)}-\mu_{\alpha \beta }\right)\right)}^{-1}=\sum_{i=1}^{k}m_{i}\lambda_{i}.$$

It follows that ${\bar{Y}}_{\alpha \beta }$ satisfies

$$\sum_{i=1}^{k}\frac{m_{i}\left({\bar{Y}}_{\alpha \beta i}-\mu_{\alpha \beta }\right)}{m_{i}^{-1}\sum_{j=q_{i}}^{r_{i}}{\left(Y_{ij}-\mu_{\alpha \beta }\right)}^{2}}=o_{p}\left(\sqrt{m_{0}}\right),$$

and expression (D3) follows. Since, according to Theorem C1, for each of the trimmed samples i=1,…,k and true value $\mu_{\alpha \beta }^{0}$,

$$-2a_{i}\log R_{i}\left(\mu_{\alpha \beta }^{0}\right)\overset{d}{\to }\chi_{1}^{2},$$

then, summing over the groups, we prove the result stated in (D3), by using the same arguments leading to the result stated in (B2). ☐
